# Supplementary material for: What do people living with chronic pain want from a pain forecast? A research prioritization study
Source: PLoS One. 2023 Oct 12;18(10):e0292968. doi: 10.1371/journal.pone.0292968 (PMC10569639; doi:10.1371/journal.pone.0292968)
Supplement: Attachment — Submitted filename: Response2Reviewers-R2.docx [file pone.0292968.s002.docx]

**Response to Editor and Reviewer Comments**

Authors’ responses in red.

PONE-D-23-13975

What do people living with chronic pain want from a pain forecast? A research prioritization study

Dear Dr. Schultz,

Thank you for submitting your manuscript to PLOS ONE. After careful consideration, we feel that it has merit but does not fully meet PLOS ONE’s publication criteria as it currently stands. Therefore, we invite you to submit a revised version of the manuscript that addresses the points raised during the review process.

Thank you for the prompt handling of our manuscript. We appreciate the time and effort that went into handling our manuscript.

Please submit your revised manuscript by Sep 22 2023 11:59PM. If you will need more time than this to complete your revisions, please reply to this message or contact the journal office at [plosone@plos.org](mailto:plosone@plos.org). When you're ready to submit your revision, log on to [https://www.editorialmanager.com/pone/ [editorialmanager.com]](https://urldefense.com/v3/__https:/www.editorialmanager.com/pone/__;!!PDiH4ENfjr2_Jw!AVe9uZ8NxKRZYNAcHHcEAhT1TMSi0IqHjYHU7LOAsm8sKsSjqX-UUQJCke7xszw-DTrnZX-vgorbb3S1YdzAv58rAf8$) and select the 'Submissions Needing Revision' folder to locate your manuscript file.

Please include the following items when submitting your revised manuscript:

- A rebuttal letter that responds to each point raised by the academic editor and reviewer(s). You should upload this letter as a separate file labeled 'Response to Reviewers'.
- A marked-up copy of your manuscript that highlights changes made to the original version. You should upload this as a separate file labeled 'Revised Manuscript with Track Changes'.
- An unmarked version of your revised paper without tracked changes. You should upload this as a separate file labeled 'Manuscript'.

These three files have been uploaded.

If you would like to make changes to your financial disclosure, please include your updated statement in your cover letter. Guidelines for resubmitting your figure files are available below the reviewer comments at the end of this letter.

The cover letter has been revised. The figures have now been passed through PACE, and the .tifs have been uploaded.

If applicable, we recommend that you deposit your laboratory protocols in protocols.io to enhance the reproducibility of your results. Protocols.io assigns your protocol its own identifier (DOI) so that it can be cited independently in the future. For instructions see: [https://journals.plos.org/plosone/s/submission-guidelines#loc-laboratory-protocols [journals.plos.org]](https://urldefense.com/v3/__https:/journals.plos.org/plosone/s/submission-guidelines*loc-laboratory-protocols__;Iw!!PDiH4ENfjr2_Jw!AVe9uZ8NxKRZYNAcHHcEAhT1TMSi0IqHjYHU7LOAsm8sKsSjqX-UUQJCke7xszw-DTrnZX-vgorbb3S1YdzA3yifb2E$). Additionally, PLOS ONE offers an option for publishing peer-reviewed Lab Protocol articles, which describe protocols hosted on protocols.io. Read more information on sharing protocols at [https://plos.org/protocols?utm_medium=editorial-email&utm_source=authorletters&utm_campaign=protocols [plos.org]](https://urldefense.com/v3/__https:/plos.org/protocols?utm_medium=editorial-email&utm_source=authorletters&utm_campaign=protocols__;!!PDiH4ENfjr2_Jw!AVe9uZ8NxKRZYNAcHHcEAhT1TMSi0IqHjYHU7LOAsm8sKsSjqX-UUQJCke7xszw-DTrnZX-vgorbb3S1YdzARxU1dvw$).

This is not relevant to our work.

We look forward to receiving your revised manuscript.

Thank you again, and we look forward to receiving your response.

Kind regards,

Shadia Hamoud Alshahrani, PhD
Academic Editor
PLOS ONE

Journal Requirements:

When submitting your revision, we need you to address these additional requirements.

1. Please ensure that your manuscript meets PLOS ONE's style requirements, including those for file naming. The PLOS ONE style templates can be found at

[https://journals.plos.org/plosone/s/file?id=wjVg/PLOSOne_formatting_sample_main_body.pdf [journals.plos.org]](https://urldefense.com/v3/__https:/journals.plos.org/plosone/s/file?id=wjVg*PLOSOne_formatting_sample_main_body.pdf__;Lw!!PDiH4ENfjr2_Jw!AVe9uZ8NxKRZYNAcHHcEAhT1TMSi0IqHjYHU7LOAsm8sKsSjqX-UUQJCke7xszw-DTrnZX-vgorbb3S1YdzAa5LVpug$) and

[https://journals.plos.org/plosone/s/file?id=ba62/PLOSOne_formatting_sample_title_authors_affiliations.pdf [journals.plos.org]](https://urldefense.com/v3/__https:/journals.plos.org/plosone/s/file?id=ba62*PLOSOne_formatting_sample_title_authors_affiliations.pdf__;Lw!!PDiH4ENfjr2_Jw!AVe9uZ8NxKRZYNAcHHcEAhT1TMSi0IqHjYHU7LOAsm8sKsSjqX-UUQJCke7xszw-DTrnZX-vgorbb3S1YdzA69Q1NJQ$)

These three files have been uploaded.

2. Thank you for stating the following financial disclosure:

"This work was supported by infrastructure support from the Centre for Epidemiology Versus Arthritis (grant number 21755; [https://www.versusarthritis.org/research/our-current-research/our-research-centres/ [versusarthritis.org]](https://urldefense.com/v3/__https:/www.versusarthritis.org/research/our-current-research/our-research-centres/__;!!PDiH4ENfjr2_Jw!AVe9uZ8NxKRZYNAcHHcEAhT1TMSi0IqHjYHU7LOAsm8sKsSjqX-UUQJCke7xszw-DTrnZX-vgorbb3S1YdzAyzmvjR0$)). TH receives funding from the Royal Society (grant number INF/R2/180067; [https://royalsociety.org/ [royalsociety.org]](https://urldefense.com/v3/__https:/royalsociety.org/__;!!PDiH4ENfjr2_Jw!AVe9uZ8NxKRZYNAcHHcEAhT1TMSi0IqHjYHU7LOAsm8sKsSjqX-UUQJCke7xszw-DTrnZX-vgorbb3S1YdzAEtN9S64$)) and the Alan Turing Institute for Data Science and Artificial Intelligence ([https://www.turing.ac.uk/ [turing.ac.uk]](https://urldefense.com/v3/__https:/www.turing.ac.uk/__;!!PDiH4ENfjr2_Jw!AVe9uZ8NxKRZYNAcHHcEAhT1TMSi0IqHjYHU7LOAsm8sKsSjqX-UUQJCke7xszw-DTrnZX-vgorbb3S1YdzA607WTfw$)). "

Please state what role the funders took in the study.  If the funders had no role, please state:

"The funders had no role in study design, data collection and analysis, decision to publish, or preparation of the manuscript."

If this statement is not correct you must amend it as needed.

Please include this amended Role of Funder statement in your cover letter; we will change the online submission form on your behalf.

We have amended the Role of Funder statement in our cover letter to include the following: "The funders had no role in study design, data collection and analysis, decision to publish, or preparation of the manuscript."

3. Thank you for stating the following in the Competing Interests section:

"WGD has received consultancy fees from Google (google.com), and DMS has received consultancy fees from Palta ([https://palta.com/ [palta.com]](https://urldefense.com/v3/__https:/palta.com/__;!!PDiH4ENfjr2_Jw!AVe9uZ8NxKRZYNAcHHcEAhT1TMSi0IqHjYHU7LOAsm8sKsSjqX-UUQJCke7xszw-DTrnZX-vgorbb3S1YdzAl2lOmqo$)), both unrelated to this work. All other authors have declared that no competing interests exist."

Please confirm that this does not alter your adherence to all PLOS ONE policies on sharing data and materials, by including the following statement: "This does not alter our adherence to  PLOS ONE policies on sharing data and materials.” (as detailed online in our guide for authors [http://journals.plos.org/plosone/s/competing-interests).  [journals.plos.org]](https://urldefense.com/v3/__http:/journals.plos.org/plosone/s/competing-interests).__;!!PDiH4ENfjr2_Jw!AVe9uZ8NxKRZYNAcHHcEAhT1TMSi0IqHjYHU7LOAsm8sKsSjqX-UUQJCke7xszw-DTrnZX-vgorbb3S1YdzAVNcOpMg$) If there are restrictions on sharing of data and/or materials, please state these. Please note that we cannot proceed with consideration of your article until this information has been declared.

Please include your updated Competing Interests statement in your cover letter; we will change the online submission form on your behalf.

We have amended the Competing Interests statement in our cover letter to include the following: “This does not alter our adherence to PLOS ONE policies on sharing data and materials.”

4. We note that you have indicated that data from this study are available upon request. PLOS only allows data to be available upon request if there are legal or ethical restrictions on sharing data publicly. For more information on unacceptable data access restrictions, please see [http://journals.plos.org/plosone/s/data-availability#loc-unacceptable-data-access-restrictions [journals.plos.org]](https://urldefense.com/v3/__http:/journals.plos.org/plosone/s/data-availability*loc-unacceptable-data-access-restrictions__;Iw!!PDiH4ENfjr2_Jw!AVe9uZ8NxKRZYNAcHHcEAhT1TMSi0IqHjYHU7LOAsm8sKsSjqX-UUQJCke7xszw-DTrnZX-vgorbb3S1YdzAR1Jxdxs$).

In your revised cover letter, please address the following prompts:

    a) If there are ethical or legal restrictions on sharing a de-identified data set, please explain them in detail (e.g., data contain potentially sensitive information, data are owned by a third-party organization, etc.) and who has imposed them (e.g., an ethics committee). Please also provide contact information for a data access committee, ethics committee, or other institutional body to which data requests may be sent.

   b) If there are no restrictions, please upload the minimal anonymized data set necessary to replicate your study findings as either Supporting Information files or to a stable, public repository and provide us with the relevant URLs, DOIs, or accession numbers. For a list of acceptable repositories, please see [http://journals.plos.org/plosone/s/data-availability#loc-recommended-repositories [journals.plos.org]](https://urldefense.com/v3/__http:/journals.plos.org/plosone/s/data-availability*loc-recommended-repositories__;Iw!!PDiH4ENfjr2_Jw!AVe9uZ8NxKRZYNAcHHcEAhT1TMSi0IqHjYHU7LOAsm8sKsSjqX-UUQJCke7xszw-DTrnZX-vgorbb3S1YdzA1vhGiA4$).

We will update your Data Availability statement on your behalf to reflect the information you provide.

The datasets generated and analysed during the current study are not publicly available as data were collected for patient and public involvement activities and consent was obtained for the sharing of anonymous quotes and aggregated data only. The data within the *Cloudy with a Chance of Pain* study is detailed health data for a national population and is both sensitive and special category data. Given the detailed nature of the dataset, it is not possible to provide a minimal de-identified dataset that retains the necessary data utility to replicate our study’s findings and be considered anonymised. Anonymisation of the Cloudy study data (whilst retaining data utility) is only possible through a combination of measures [i.e., de-identification, data minimisation related to the use case and the provision of access via a Secure Data Environment (SDE)]. These measures are in line with the UK Anonymisation Network guidance (Elliot, Mackey, & O’Hara, 2020). We are currently working towards establishing the processes for supporting access and sharing via an SDE and anticipate the data will be more widely sharable sometime in 2024. Elaine Mackey is the contact for dataset access ([elaine.mackey@manchester.ac.uk](mailto:elaine.mackey@manchester.ac.uk)).

Elliot, M., Mackey, E., & O'Hara, K. (2020). *The Anonymisation Decision Making Framework: European Practitioners' Guide (2nd edition)*. UK Anonymisation Network. <https://msrbcel.files.wordpress.com/2020/11/adf-2nd-edition-1.pdf>.

5. Please review your reference list to ensure that it is complete and correct. If you have cited papers that have been retracted, please include the rationale for doing so in the manuscript text, or remove these references and replace them with relevant current references. Any changes to the reference list should be mentioned in the rebuttal letter that accompanies your revised manuscript.

We have reviewed the reference list for completeness and correctness. We have added one new citation and removed one.

If you need to cite a retracted article, indicate the article’s retracted status in the References list and also include a citation and full reference for the retraction notice.

To our knowledge, none of our references have been retracted.

Reviewers' comments:

Reviewer's Responses to Questions

We thank the two reviewers for their comments that have helped improve our manuscript. We thank you for your time and effort.

**Comments to the Author**

1. Is the manuscript technically sound, and do the data support the conclusions?

The manuscript must describe a technically sound piece of scientific research with data that supports the conclusions. Experiments must have been conducted rigorously, with appropriate controls, replication, and sample sizes. The conclusions must be drawn appropriately based on the data presented.

Reviewer #1: No

Reviewer #2: Yes

2. Has the statistical analysis been performed appropriately and rigorously?

Reviewer #1: No

Reviewer #2: Yes

3. Have the authors made all data underlying the findings in their manuscript fully available?

The [PLOS Data policy [plosone.org]](https://urldefense.com/v3/__http:/www.plosone.org/static/policies.action*sharing__;Iw!!PDiH4ENfjr2_Jw!AVe9uZ8NxKRZYNAcHHcEAhT1TMSi0IqHjYHU7LOAsm8sKsSjqX-UUQJCke7xszw-DTrnZX-vgorbb3S1YdzA2-ZqxiQ$) requires authors to make all data underlying the findings described in their manuscript fully available without restriction, with rare exception (please refer to the Data Availability Statement in the manuscript PDF file). The data should be provided as part of the manuscript or its supporting information, or deposited to a public repository. For example, in addition to summary statistics, the data points behind means, medians and variance measures should be available. If there are restrictions on publicly sharing data—e.g. participant privacy or use of data from a third party—those must be specified.

Reviewer #1: No

Reviewer #2: Yes

4. Is the manuscript presented in an intelligible fashion and written in standard English?

PLOS ONE does not copyedit accepted manuscripts, so the language in submitted articles must be clear, correct, and unambiguous. Any typographical or grammatical errors should be corrected at revision, so please note any specific errors here.

Reviewer #1: Yes

Reviewer #2: Yes

5. Review Comments to the Author

Please use the space provided to explain your answers to the questions above. You may also include additional comments for the author, including concerns about dual publication, research ethics, or publication ethics. (Please upload your review as an attachment if it exceeds 20,000 characters)

Reviewer #1: The article needs major enhancement especially for data presentation, results, intext referencing, major work needs to be done, perhapse rewriting at this point and resubmit it again.

We appreciate the time that you spent reading our manuscript. These comments do not appear to identify the specific concerns that you have with our manuscript, as such we hope we have addressed your concerns.

We can document the changes that we made based on the comments of Reviewer #2 and our own revisions to improve the manuscript.

- The abstract has more details about the specific methods employed.
- We have added another reference in the introduction about the impact of chronic pain globally.
- We have moved the methods section to its more traditional location in between the introduction and results. We hope this move will better facilitate the readability of the manuscript.
- The methods section has been enhanced with more details requested by Reviewer #2.
- The results section has been enhanced by adding the four tables and accompanying analysis from the former Supplemental Material to the manuscript, thereby adding more substantive results to the manuscript, as requested.
- We have added a new conclusion section to ensure that the take-home messages are clear to the reader.

Also you need to focus on presenting the data in more than a percentage.

This is a fair point. We have now added the number of survey respondents in parentheses in addition to the percentages throughout the entire manuscript. We think it makes the reading a bit more dense, but we have adopted your suggestion, nevertheless.

Reviewer #2: Overall, it is a unique study and gives scientific insight into the fraternity on the development of the pain forecast model.

Thank you for your kind words on our manuscript. We appreciate your efforts to provide these comments that have helped improve our manuscript. Thank you.

1. Title: The title of the manuscript is appropriate.

Thank you.

2. Introduction: In line no. 36, 37, It would be better to include the most recent references which will give more strength to the need for the study.

As we write in the manuscript, “up-to-date figures are scarce”. We looked quite hard before the original submission—and again during this revision—to see if we could find more recent estimates of the economic impacts of pain. We could find none more recent than 2012. As the incidences of pain change very little with time (Chen et al. 2022), we see little problem citing numbers from 10–12 years ago, as is typically done in other recently published studies that have also used these same numbers that we have cited.

Nevertheless, to accommodate the reviewer’s comment as best as we can, we have deleted the reference to the study using 1998 data. Also, we have added a citation to 568 million global cases of lower-back pain from a 2022 study using 2019 data. Perhaps it is time for pain researchers to recalculate the economic impacts of chronic pain using more recent data!

3. Study Design: It is unclear from the abstract what is the type and design. It would be helpful to clarify the study design and approach.

We have now added further language about the study design, which comprises two PPI activities: a focus group and a survey. Thank you for this suggestion.

4. Methods Section: The methods section lacks important details regarding the study sample selection.

To facilitate the readability of the manuscript, we have moved the methods section describing the focus group and survey to its more traditional place immediately after the introduction and before the results section.

• Is there any matching done according to the sample characteristics

No matching was performed. We have added a statement in the manuscript attesting to this fact.

• How the survey question was validated

No formal validation on the survey questions were performed. More generally, however, these questions and the answer selections on the survey arose from discussions within the focus group. That should have helped ensure that the questions were informed by conversations with people living with chronic pain. We have added a statement attesting to this fact in the methods section.

• In which way, the survey was questionnaire was administered

The survey was created in Qualtrics, and a link was shared on social media and with the charities listed in Table 1. We have added this information to the revised manuscript.

Please clearly the above quarries.

Thank you for these comments. We hope our revisions suitably improve the manuscript.

5. Analysis of data: It is unclear how the data was analyzed. Kindly elaborate on it.

By moving the methods section into the body of the text in between the introduction and the results, it should be more clear to the readers what our methods were for the analysis of the data. We hope this addresses your concern.

7. Conclusion Section: Consider discussing the implications of the study's findings.

We have now added a traditional conclusion section that summarizes the results of the study and discusses the implications of our findings.

6. PLOS authors have the option to publish the peer review history of their article ([what does this mean? [journals.plos.org]](https://urldefense.com/v3/__https:/journals.plos.org/plosone/s/editorial-and-peer-review-process*loc-peer-review-history__;Iw!!PDiH4ENfjr2_Jw!AVe9uZ8NxKRZYNAcHHcEAhT1TMSi0IqHjYHU7LOAsm8sKsSjqX-UUQJCke7xszw-DTrnZX-vgorbb3S1YdzAZb6un8Y$)). If published, this will include your full peer review and any attached files.


If you choose “no”, your identity will remain anonymous but your review may still be made public.


**Do you want your identity to be public for this peer review?** For information about this choice, including consent withdrawal, please see our [Privacy Policy [plos.org]](https://urldefense.com/v3/__https:/www.plos.org/privacy-policy__;!!PDiH4ENfjr2_Jw!AVe9uZ8NxKRZYNAcHHcEAhT1TMSi0IqHjYHU7LOAsm8sKsSjqX-UUQJCke7xszw-DTrnZX-vgorbb3S1YdzA1tjRuoc$).

Reviewer #1: No

Thank you, Reviewer #1.

Reviewer #2: **Yes:** PREMALATHA PAULSAMY

Thank you, Dr. Paulsamy.

[NOTE: If reviewer comments were submitted as an attachment file, they will be attached to this email and accessible via the submission site. Please log into your account, locate the manuscript record, and check for the action link "View Attachments". If this link does not appear, there are no attachment files.]

While revising your submission, please upload your figure files to the Preflight Analysis and Conversion Engine (PACE) digital diagnostic tool, [https://pacev2.apexcovantage.com/ [pacev2.apexcovantage.com]](https://urldefense.com/v3/__https:/pacev2.apexcovantage.com/__;!!PDiH4ENfjr2_Jw!AVe9uZ8NxKRZYNAcHHcEAhT1TMSi0IqHjYHU7LOAsm8sKsSjqX-UUQJCke7xszw-DTrnZX-vgorbb3S1YdzAzJZTrBs$). PACE helps ensure that figures meet PLOS requirements. To use PACE, you must first register as a user. Registration is free. Then, login and navigate to the UPLOAD tab, where you will find detailed instructions on how to use the tool. If you encounter any issues or have any questions when using PACE, please email PLOS at [figures@plos.org](mailto:figures@plos.org). Please note that Supporting Information files do not need this step.

We have passed our two figures through PACE, producing .tif format figures that have now been uploaded with the manuscript.
